# ANGPTL3 in the Peripheral Circulation Is Associated with Resistance to Anti-PD1 Therapy in Advanced Gastric Cancer

**DOI:** 10.1158/2767-9764.CRC-25-0793

**Published:** 2026-02-19

**Authors:** Chie Kudo-Saito, Hirokazu Shoji, Kengo Nagashima, Hiroshi Imazeki, Kai Tsugaru, Naoki Takahashi, Takeshi Kawakami, Yusuke Amanuma, Takeru Wakatsuki, Naohiro Okano, Yukiya Narita, Yoshiyuki Yamamoto, Rika Kizawa, Kei Muro, Narikazu Boku

**Affiliations:** 1 Department of Immune Medicine, National Cancer Center Research Institute, Tokyo, Japan.; 2 Department of Gastrointestinal Medical Oncology, National Cancer Center Hospital, Tokyo, Japan.; 3 Biostatistics Unit, Clinical and Translational Research Center, Keio University Hospital, Tokyo, Japan.; 4 Division of Gastroenterology and Hepatology, Keio University Hospital, Tokyo, Japan.; 5 Department of Gastroenterology, Saitama Cancer Center, Saitama, Japan.; 6 Division of Gastrointestinal Oncology, Shizuoka Cancer Center, Shizuoka, Japan.; 7 Clinical Trial Promotion Department, Chiba Cancer Center, Chiba, Japan.; 8 Department of Gastrointestinal Medical Oncology, Cancer Institute Hospital of JFCR, Tokyo, Japan.; 9 Department of Medical Oncology, Kyorin University Faculty of Medicine, Tokyo, Japan.; 10 Department of Clinical Oncology, Aichi Cancer Center, Nagoya, Japan.; 11 Department of Gastroenterology, University of Tsukuba Hospital, Tsukuba, Japan.; 12 Department of Medical Oncology, Toranomon Hospital, Tokyo, Japan.; 13 Department of Medical Oncology and General Medicine, IMS Hospital, Institute of Medical Science, University of Tokyo, Tokyo, Japan.

## Abstract

Anti-PD1/-PDL1 therapy has attracted great attention in cancer therapy in recent years, but a serious problem remains that only a small portion of patients can benefit from it. In this study, we attempted to identify a molecule that is associated with anti-PD1/-PDL1 therapeutic efficacy through proteomic profiling of plasma obtained from patients with advanced gastric cancer (AGC) receiving anti-PD1 nivolumab monotherapy. We collected peripheral blood from 91 patients with AGC before and after nivolumab treatment, and plasma was analyzed by the SomaScan v4.1 and ELISA. Relationships between the levels and patient prognosis were statistically analyzed. To evaluate antitumor effects induced by blocking the identified molecule for which high levels were significantly associated with poor prognosis, *in vivo* therapeutic experiments using mouse tumor models were conducted. Proteomic data revealed that the levels of 14 molecules both before and after treatment were significantly higher in patients with progressive disease (PD) than those in non-PD patients. Among them, angiopoietin-like 3 (ANGPTL3) levels were notably higher in PD patients than those in non-PD patients, and patients with high levels of ANGPTL3 either before or after treatment showed significantly worse prognosis. In mouse tumor models with increased ANGPTL3, anti-ANGPTL3 therapy significantly reduced tumor growth and synergistically enhanced anti-PD1 therapeutic efficacy. These suggest that high levels of ANGPTL3 in plasma are a significant risk factor associated with unresponsiveness to anti-PD1 treatment and poor prognosis. Targeting ANGPTL3 in the peripheral circulation may be a promising strategy to improve clinical outcomes in anti-PD1/-PDL1 therapy for AGC.

**Significance:**

This study provides valuable evidence suggesting the importance of targeting peripheral ANGPTL3 in the anti-PD1/-PDL1 therapy for AGC and will encourage and accelerate the development of a useful biomarker to predict responders/nonresponders to the therapy, leading to improved clinical outcomes in the treatment of gastric cancer.

## Introduction

Gastric cancer is the fifth most common type of cancer and the fifth leading cause of cancer-related deaths worldwide, and unresectable advanced/recurrent gastric cancer or gastroesophageal junction cancer (abbreviated as AGC) is rarely curable even by multimodal treatment with surgery, chemotherapy, and radiotherapy (1). New immunotherapeutics to block immune checkpoint pathways mediated by specific molecules, such as PD1, PDL1, and CTLA4, have brought great advances in cancer treatment and have been providing a treatment option for patients with advanced cancers of various types, including gastric cancer (2). However, anti-PD1/-PDL1 therapy still has many issues, including a relatively low response rate of approximately 15% (3, 4), immune-related adverse events (5), and high medical costs (6). Therefore, there is an urgent need to identify biomarkers to predict potential responders/nonresponders before starting it and to establish combination therapies that can synergistically enhance the therapeutic efficacy in clinical practice.

To date, numerous challenges have been undertaken around the world using advanced technologies to analyze tumor tissues and peripheral blood collected from patients with cancer receiving anti-PD1/-PDL1 therapy, and many factors related to the effectiveness of anti-PD1/-PDL1 therapy have been identified. For example, proteomic analysis has been widely used around the world as it can detect a variety of proteins with posttranslational modifications, which are involved in various physiologic functions and diseases (7). However, the results differ depending on the sampling method and data analytic method. For example, some proteins in serum are degraded during coagulation reaction, whereas plasma treated with anticoagulants has relatively little effect on the quality and quantity of proteins and retains more protein (8). In gastric cancer, several small-scale (9) and large-scale proteomic analyses (10, 11) using patient plasma identified many biomarkers (CNDP1, DEK, LY6D, and SLURP1) that are significantly associated with tumor stages and patient prognosis. The REGOMUNE phase II trial identified several plasma cytokines (CSF1, IL4, IL8, and TWEAK) that were significantly associated with poor prognosis in patients receiving anti-PDL1 avelumab, in combination with a multi–tyrosine kinase inhibitor regorafenib (12). However, the combination of two drugs complicates interpretation of the data. Deep understanding of single-drug effects will be paramount to identify biomarkers that more accurately predict responsiveness to anti-PD1/-PDL1 therapy and develop drugs to enhance their effects more synergistically.

To address these issues, in this study, we first conducted proteomic analysis of plasma isolated from peripheral blood collected from patients with AGC before and after anti-PD1 nivolumab monotherapy and statistically analyzed the relationship with patient prognosis. Secondarily, to clarify the significance of targeting the molecule identified in the clinical study, we conducted *in vivo* therapeutic experiments using mouse tumor models.

## Materials and Methods

### Patients

The WJOG10417GTR study (UMIN000032686) was collaboratively conducted in 10 hospitals according to the protocol (August 2018–November 2020) approved by the Institutional Review Board of each participating hospital, including the National Cancer Center (no. 2017-473 and no. 2021-144). Written informed consent was obtained from all patients before study enrollment. Ninety-six patients with AGC (67 males, 29 females, and median age of 69 years) received intravenous infusions of anti-PD1 mAb nivolumab (RRID: AB_3694346) at 3 mg/kg or 240 mg/body every 2 weeks until disease progression or unacceptable toxicity. The median follow-up period was 21 months. The details of the WJOG10417GTR study, including eligibility, tumor responses, and toxicities, were previously reported (13). All activities were conducted in accordance with the ethical principles of the Declaration of Helsinki and the Japanese Clinical Research Ethics Guidelines.

### Sample preparation

EDTA-added peripheral blood was collected from patients before and 1 month after nivolumab monotherapy as the immune status agitated by treatment was considered to subside within the patient’s body approximately 1 month later [after 21 days or more (22–43 days) from the first drug administration], as described before (13). Peripheral blood could not be collected from five patients before treatment and from 16 patients after treatment because of poor condition. Plasma was separated from peripheral blood by high-speed centrifugation.

### Proteomic profiling of plasma

The levels of multiple proteins in the plasma obtained before (*n* = 88) and after treatment (*n* = 80) were measured using the SomaScan 7K Assay v4.1 (SomaLogic Inc.), which is an aptamer-based proteomics assay system capable of measuring 7,288 human proteins. Three patients with insufficient plasma volume collected before treatment were excluded from proteomic analysis. The detected protein levels were indicated as relative fluorescence units (RFU).

### ELISA

Human angiopoietin-like 3 (ANGPTL3) protein concentrations in the plasma obtained before (*n* = 91) and after treatment (*n* = 80) were measured using an ELISA kit (R&D Systems, #DANL30) according to the manufacturer’s instructions.

### Mice and cell lines

Five-week-old female BALB/c mice (RRID: MGI:2161072) were purchased from Charles River Laboratories and were maintained under pathogen-free conditions. The mice were used according to the protocols (no. T17-055) approved by the Animal Care and Use Committee at the National Cancer Center Research Institute. Murine colon cancer Colon26 cells (i.e., MC-26; RRID: CVCL_0240) were purchased from Cell Resource Center for Biomedical Research at Tohoku University in Japan. Cells were assessed for *Mycoplasma* negativity using a Hoechst staining detection kit (MP Biomedicals) and were expanded and frozen in liquid nitrogen to avoid changes occurred by a long-term culture before use in experiments. Cells were cultured in 10% FBS–containing DMEM (Gibco).

### *In vivo* therapy

BALB/c mice were subcutaneously implanted with Colon26 tumor cells (5 × 10^5^) and received intraperitoneal injection with anti-ANGPTL3 mAb (Ichorbio, #ICH5151; RRID: AB_3075795), anti-PD1 mAb (clone 29F.1A12; Bio X Cell, #BE0273), or mouse IgG (mIgG; clone MOPC-21; Bio X Cell, #BE0083) as a control at 10 mg/kg on days 3 and 10 after tumor implantation (*n* = 5–10 per group by randomization). The subcutaneous tumor volume (0.5 × length × width^2^, mm^3^) was measured every 2 to 3 days. On day 17 to 20, the subcutaneous tumors and spleens were harvested from the mice, and the isolated cells were analyzed by flow cytometry. To assess tumor-killing activity of CD8^+^ T cells, bulk spleen cells (SPC) were prestimulated with the H-2L(d)–restricted tumor antigen AH1 peptide (1 µg/mL; MBL, #TS-M521-P) for 6 days, and the recovered CD8^+^ T cells were tested for cytotoxic activity (4 hours) as described before (14). Cytotoxic activity of NK cells was similarly assessed using Yac-1 cells as a target. Only the leader had complete information, and the experiments were conducted under blind conditions.

### Flow cytometric analysis

After Fc blocking, tumor-infiltrating lymphocytes and SPCs obtained from mice were stained with the following immunofluorescence-conjugated antibodies: anti-CD45-PE-Cy7 (BioLegend, #103114; RRID: AB_312979), anti-CD3e-BUV496 (BD Biosciences, #612955; RRID: AB_2870231), anti-CD4-BV785 (BioLegend, #100552; RRID: AB_2563053), anti-CD8a-BUV395 (BD Biosciences, #563786; RRID: AB_2732919), anti-DX5/CD49b-APC-Cy7 (BioLegend, #108920; RRID: AB_2561458), anti-CD11b-BV711 (BioLegend, #101242; RRID: AB_2563310), anti-F4/80-BV510 (BioLegend, #123135; RRID: AB_2562622), anti-CD11c-BV650 (BioLegend, #117339; RRID: AB_2562414), anti-Ki67-FITC (BioLegend, #652410; RRID: AB_2562141), anti-GZMB-Pacific Blue (BioLegend, #515408; RRID: AB_2562196), AH1-tetramer-PE (MBL, #TS-M521-1), anti-Foxp3-BV421 (BioLegend, #126419; RRID: AB_2565933), anti-TIGIT-PE-Cy7 (BioLegend, #142108; RRID: AB_2565649), and the appropriate isotype control. For intracellular staining, cells were treated with Cytofix/Cytoperm solution (BD Biosciences) before antibody staining. Data were acquired using a BD LSRFortessa X-20 cytometer (RRID: SCR_013311) and were analyzed by FlowJo software (BD Biosciences; RRID: SCR_008520). Cells were first gated by FSC-A/SSC-A to exclude debris and then by CD45 to define leukocytes before analyzing specific molecular expression compared with isotype controls.

### Statistical analysis

Significant differences (*P* value < 0.05) were statistically evaluated using GraphPad Prism 7 (MDF; RRID: SCR_002798), SAS software version 9.4 (RRID: SCR_008567), and R (RRID: SCR_001905) version 4.2.3. Data were analyzed by one-way or two-way ANOVA with Bonferroni *post hoc* test for pairwise comparison of multiple groups on the basis of the normal distributions. The normality of the dataset was analyzed using the Shapiro–Wilk test, and the dataset showing a nonnormal distribution was analyzed using the Mann–Whitney U test. Progression-free survival (PFS) and overall survival (OS) were estimated using the Kaplan–Meier method. Hazard ratios (HR) were obtained by univariable and multivariable models adjusting potential confounding factors [propensity score, prior gastrectomy, number of metastatic organs, and alkaline phosphatase (ALP)], and 95% confidence intervals were calculated. Cutoff values were determined by change point of log HRs using the Cox regression models with penalized splines, and we used the cutoff values with the larger HRs calculated through careful analysis of each marker based on the method for modeling continuous-scale covariates according to a standard survival analysis textbook, as described before (13).

## Results

### ANGPTL3 levels are high in the peripheral blood of patients with AGC who progress after nivolumab treatment

First, we compared the proteomic profiling data among three groups based on treatment response patterns although caution is needed when interpreting the results as the small number of *n* of the non–progressive disease (PD) group limits the statistical reliability and generalizability of the results to clinical practice because of the potential biases and uncertainties arising from the small sample size even if statistical significance was obtained: one showed PD within 1 month after treatment (early PD group), one showed PD more than 1 month after treatment (late PD group), and one did not show PD at least during the study period (non-PD group). Mean RFU levels of 14 proteins both before and after treatment were significantly higher in early/late PD groups as compared with those in the non-PD group, and posttreatment levels of ANGPTL3 were particularly notable, being more than 30-fold higher in the early PD group than those in the non-PD group (Supplementary Fig. S1). These suggest that the 14 proteins, especially ANGPTL3, are involved in unresponsiveness to nivolumab therapy.

**Figure 1.**
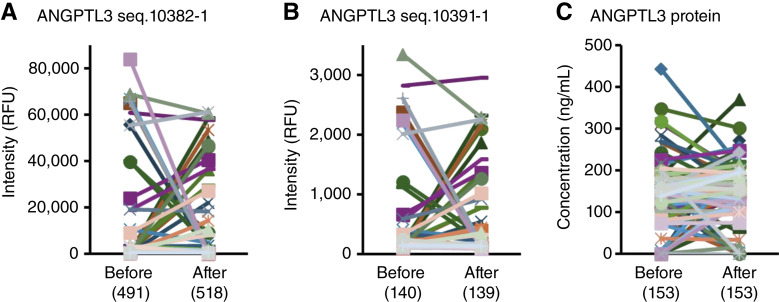
ANGPTL3 levels are high in the peripheral blood of patients with AGC who progress after nivolumab treatment. Peripheral blood was collected from patients with AGC before and 1 month after initiating nivolumab therapy, and ANGPTL3 levels in plasma were measured by proteomic profiling or ELISA (*n* = 80). **A,** ANGPTL3 seq.10382-1 detected by proteomic profiling. **B,** ANGPTL3 seq.10391-1 detected by proteomic profiling. **C,** ANGPTL3 protein detected by ELISA. The median values for each group are shown in brackets. No significant differences were observed between pretreatment and posttreatment levels for any of the markers.

ANGPTL3 plays a key role in lipid metabolism, specifically by inhibiting lipoprotein lipase, which is essential for the clearance of triglyceride-rich lipoproteins from the bloodstream, thereby increasing lipid accumulation in the blood (15). The proteomic data contained two ANGPTL3 SeqIDs, seq.10382-1 (Fig. 1A) and seq.10391-1 (Fig. 1B), and levels of both at before and after treatment were significantly correlated, despite the former having much higher levels (*P* < 0.001; Supplementary Fig. S2). We next verified ANGPTL3 protein concentrations in plasma by ELISA (Fig. 1C). However, there was no correlation between the ELISA and proteomic data, and rather a significant inverse correlation was seen at baseline (*P* < 0.02; Supplementary Fig. S2). The exact reasons are unclear, and they will be discussed in detail later. For all markers, no significant difference was observed between pretreatment and posttreatment levels.

### High plasma ANGPTL3 levels are significantly associated with poor prognosis of patients with AGC after nivolumab treatment

**Figure 2.**
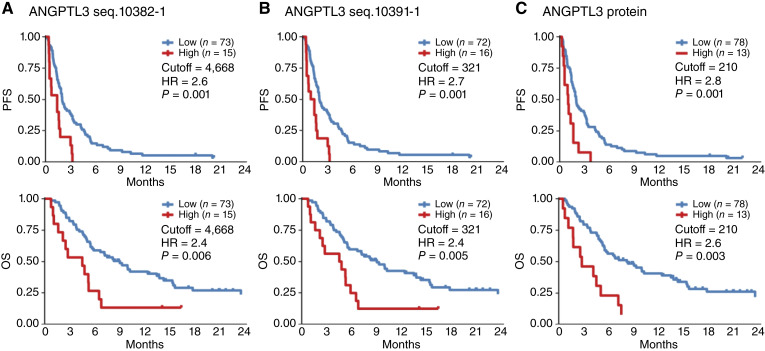
High plasma ANGPTL3 levels are significantly associated with poor prognosis of patients with AGC after nivolumab treatment. Patients were divided into two groups, high (red lines) and low (blue lines), based on ANGPTL3 cutoff values before treatment, and significant differences in PFS and OS were statistically analyzed by the Kaplan–Meier method and the Mantel–Cox log-rank test. **A,** ANGPTL3 seq.10382-1 detected by proteomic profiling. **B,** ANGPTL3 seq.10391-1 detected by proteomic profiling. **C,** ANGPTL3 protein detected by ELISA.

Next, patients were divided into two groups based on cutoff values determined by change point of log HRs using the Cox regression models with penalized splines as described before (13), and significant differences in PFS and OS were statistically analyzed. High baseline levels of any markers were significantly associated with poor prognosis: for seq.10382-1, the median PFS (mPFS) was 1.4 versus 2 months, with HR = 2.6 and *P* = 0.001 and the median OS (mOS) was 4.5 versus 9 months, with HR = 2.4 and *P* = 0.006; for seq.10391-1, mPFS was 1.2 versus 2 months, with HR = 2.7 and *P* = 0.001 and mOS was 4.6 versus 9 months, with HR = 2.4 and *P* = 0.005; and for ELISA of ANGPTL3, mPFS was 1 versus 2 months, with HR = 2.8 and *P* = 0.001 and mOS was 2.8 versus 8.1 months, with HR = 2.6 and *P* = 0.003 (Fig. 2; Table 1).

**Table 1.** Summary of PFS and OS of the two groups divided by the cutoff values of ANGPTL3.

Time points	ANGPTL3	Groups (*n*)	PFS (95% CI)	OS (95% CI)
Before treatment	Seq.10382-1	Low (73)	2 (1.7–3.1)	9 (5.8–14.3)
	​	High (15)	1.4 (0.5–3)	4.5 (2.2–6.8)
	Seq.10391-1	Low (72)	2 (1.7–3.2)	9 (5.7–14.3)
	​	High (16)	1.2 (0.5–3)	4.6 (2.2–6.8)
	Protein	Low (78)	2 (1.6–3)	8.1 (5.5–13.7)
	​	High (13)	1 (0.6–NA)	2.8 (1.7–NA)
After treatment	Seq.10382-1	Low (61)	2.6 (1.9–3.5)	9.9 (6.7–15.6)
	​	High (19)	1.3 (1–2)	4.5 (2.8–NA)
	Seq.10391-1	Low (54)	3 (2–4.3)	12.4 (8.1–23.5)
	​	High (26)	1.4 (0.9–2)	4.2 (2.8–6.8)
	Protein	Low (69)	2 (1.8–3.3)	9 (5.8–15.3)
	​	High (11)	1.5 (1.3–NA)	4.9 (4.1–NA)

Abbreviations: 95% CI, 95% confidence interval; NA, not available.

**Figure 3.**
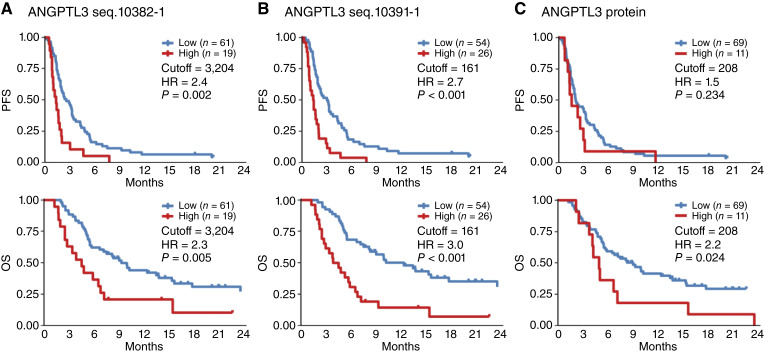
High posttreatment ANGPTL3 levels are significantly associated with shorter OS of patients with AGC after nivolumab treatment. Patients were divided into two groups, high (red lines) and low (blue lines), based on ANGPTL3 cutoff values after treatment, and significant differences in PFS and OS were statistically analyzed by the Kaplan–Meier method and the Mantel–Cox log-rank test. **A,** ANGPTL3 seq.10382-1 detected by proteomic profiling. **B,** ANGPTL3 seq.10391-1 detected by proteomic profiling. **C,** ANGPTL3 protein detected by ELISA.

Posttreatment outcomes were similar to baseline results, with both proteomic molecules significantly associated with poor prognosis, whereas ELISA data were only significantly associated with shorter OS: for seq.10382-1, mPFS was 1.3 versus 2.6 months, with HR = 2.4 and *P* = 0.002 and mOS was 4.5 versus 9.9 months, with HR = 2.3 and *P* = 0.005; for seq.10391-1, mPFS was 1.4 versus 3 months, with HR = 2.7 and *P* < 0.001 and mOS was 4.2 versus 12.4 months, with HR = 3 and *P* < 0.001; and for ELISA of ANGPTL3, mOS was 4.9 versus 9 months, with HR = 2.2 and *P* = 0.024 (Fig. 3; Table 1). These suggest that high plasma ANGPTL3 levels are a significant poor prognostic factor in nivolumab therapy for AGC.

High levels of each molecule were significantly associated with several patient characteristics, but not with established biomarkers such as combined positive score of PDL1, as summarized in Supplementary Table S1. At baseline, seq.10382-1 was associated with negative lymph node metastasis (*P* = 0.038) and all data with sex/male (seq.10382-1, *P* = 0.023; seq.10391-1, *P* = 0.010; and ELISA of ANGPTL3, *P* = 0.045); at posttreatment time point, both proteomic data were associated with negative lymph node metastasis (seq.10382-1, *P* = 0.012; seq.10391-1, *P* = 0.028), seq.10391-1 was associated with high ALP (≥350 U/L; *P* = 0.032), and ELISA of ANGPTL3 was associated with high neutrophil to lymphocyte ratio (≥1.93; *P* = 0.020). These imply that high ANGPTL3 levels may be linked to inflammatory responses in patient bodies, especially female patients, although the reason for their association with negative lymph node metastasis is unclear.

### Blocking ANGPTL3 synergistically enhances anti-PD1 therapeutic efficacy in mouse tumor models

Clinical data suggest that ANGPTL3 may be a promising molecule as a biomarker for predicting anti-PD1 responses and/or as a target for therapeutic drugs to enhance anti-PD1 therapeutic efficacy. Then, we tested this possibility using mouse tumor models implanted with murine colorectal cancer Colon26 or MC38 cells because we were unable to obtain mouse gastric cancer cell lines. Open databases, such as ProteinAtlas (https://www.proteinatlas.org/), show that human tumor cell lines, except for liver cancer cell lines, do not express ANGPTL3 in a wide range of cancer types, including gastric cancer. We also confirmed by ELISA that ANGPTL3 is not detected in the culture supernatant of human gastric cancer cell lines, such as KATO III and NCI-N87, and mouse tumor cell lines used in the *in vivo* experiments, suggesting no production of ANGPTL3 (Supplementary Fig. S3A). In the peripheral blood of mice, however, high levels of ANGPTL3 were observed even in naïve mice, and the levels were further elevated by tumor implantation (*P* < 0.001; Supplementary Fig. S3A and S3B). As ANGPTL3 is a protein secreted mainly from the liver, livers were harvested from mice, and the ANGPTL3 concentration in the extract solution was also measured by ELISA. Similar to the serum data, ANGPTL3 concentration in livers of tumor-implanted mice was significantly higher than that of naïve mice (*P* < 0.001; Supplementary Fig. S3A). These suggest that ANGPTL3 circulating in the peripheral blood originates from host tissues (presumably the liver), and its production is further enhanced by the presence of tumors within the host, possibly acting as a form of stress.

**Figure 4.**
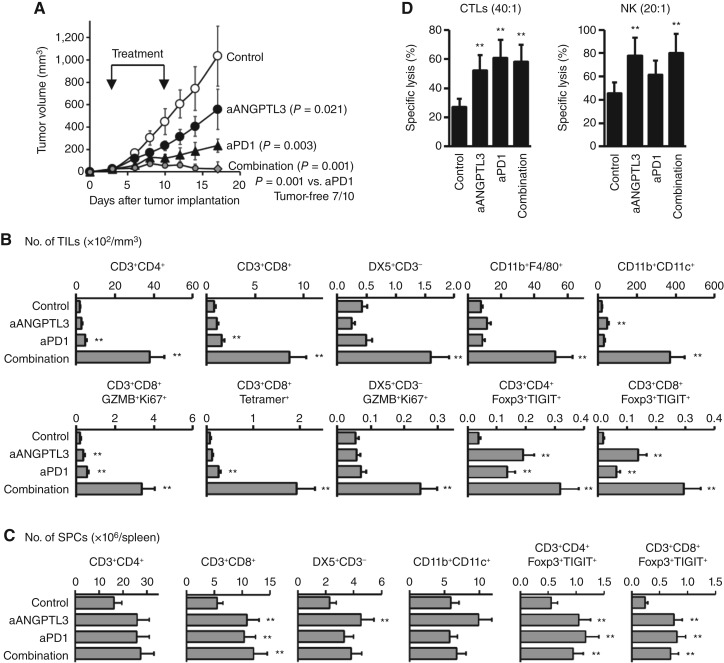
Blocking ANGPTL3 synergistically enhances anti-PD1 therapeutic efficacy in mouse tumor models. BALB/c mice were subcutaneously (5 × 10^5^) implanted with Colon26 cells, and received intraperitoneal injection of anti-ANGPTL3 mAb, anti-PD1 mAb, or mIgG as a control at 10 mg/kg on days 3 and 10 after tumor implantation (*n* = 5). On day 17, the subcutaneous tumor and spleen were harvested randomly from three of 10 mice for assays. **A,** Suppression of tumor growth by treatments (*n* = 10). Open circles, mIgG control; closed circles, anti-ANGPTL3 mAb; and closed triangles, anti-PD1 mAb. Gray diamonds, anti-ANGPTL3 mAb and anti-PD1 mAb. **B,** Dramatical increase of tumor-infiltrating lymphocytes (TIL) by anti-ANGPTL3/-PD1 combination therapy. Tumor-infiltrating lymphocytes were analyzed by flow cytometry (*n* = 3). **C,** Increase of SPCs by treatments. SPCs were analyzed by flow cytometry (*n* = 3). **D,** Enhancement of the cytotoxic activity of splenic CD8^+^ T cells by anti-ANGPTL3/-PD1 combination therapy. AH1-prestimulated CD8^+^ T cells were cocultured with Colon26 cells as a target at effector:target (E:T) ratio = 40:1 for 4 hours (*n* = 3). Splenic DX5^+^ NK cells were also cocultured with Yac1 cells as a target at E:T ratio = 20:1 for 4 hours (*n* = 3). Graphs show means ± SDs. **, *P* < 0.05 vs. mIgG control analyzed by the Mann–Whitney U test, two-way ANOVA, or one-way ANOVA. Representative data of an experiment of three independent experiments with consistent results. aANGPTL3, anti-ANGPTL3; aPD1, anti-PD1.

We next treated mice with inhibitory mAbs specific for ANGPTL3 or PD1 (10 mg/kg) on days 3 and 10 after tumor implantation. Anti-ANGPTL3 treatment significantly suppressed tumor growth (*P* = 0.003 vs. control group) although the antitumor efficacy was much lower than that of anti-PD1 treatment (*P* = 0.019, Fig. 4A). ANGPTL3 levels in the peripheral blood of anti–ANGPTL3-treated mice were slightly but significantly reduced to the level of naïve mice (*P* < 0.001) although such reduction was not seen in anti–PD1-treated mice (Supplementary Fig. S3B). The combination of both treatments significantly enhanced each monotherapeutic efficacy (*P* = 0.019 vs. anti-ANGPTL3; *P* = 0.001 vs. anti-PD1), and subcutaneous tumors disappeared in 70% of mice on day 17 after tumor implantation. When these mice were rechallenged with the same tumor cells, tumors failed to engraft and were rejected, suggesting induction of immunologic memory in the mice. Even in other, more severe models with ascites or lung metastasis, anti-ANGPTL3 therapy induced significant antitumor effects and synergistically enhanced the anti-PD1 therapeutic efficacy (Supplementary Fig. S4). These suggest that blocking ANGPTL3 may be suitable for optimizing anti-PD1 therapeutic efficacy synergistically. In the mice for which tumors disappeared, peripheral ANGPTL3 levels were significantly lower as compared with those in the mIgG-treated control mice, whereas the levels were still high in mice that still had small tumors (Supplementary Fig. S3C). This suggests that low ANGPTL3 levels in the peripheral circulation are associated with anti-PD1 responses leading to complete cure, highlighting the importance of ANGPTL3 control in anti-PD1 therapy.

Unlike either monotherapy, anti-ANGPTL3/-PD1 combination therapy significantly and dramatically induced infiltration of large numbers of antitumor effector cells, including CD3^+^ T cells, DX5^+^ NK cells, and CD11c^+^ dendritic cells, into subcutaneous tumors although there was also a marked increase in the infiltration of Foxp3^+^TIGIT^+^ T cells and F4/80^+^ macrophages, which may possibly induce immunosuppressive and inflammatory responses (Fig. 4B; Supplementary Fig. S5). Within the spleen, however, various immune cell populations similarly increased in all mAb-treated mice (Fig. 4C), and the cytotoxic activity of the splenic T cells and NK cells was similarly enhanced (Fig. 4D), with no differences observed between treatment groups. Collectively, our data revealed that hosts (including patients with AGC) with high plasma ANGPTL3 levels are unlikely to benefit from PD1 blockade therapy and that ANGPTL3 blockade therapy may be able to successfully optimize anti-PD1 therapeutic efficacy.

## Discussion

We conducted proteomic profiling of multiple proteins in plasma collected from patients with AGC before and after nivolumab monotherapy and found that high plasma ANGPTL3 levels are a significant risk factor associated with poor prognosis in patients with AGC after nivolumab therapy. In addition, based on the clinical data, we conducted *in vivo* therapeutic experiments using mouse tumor models with increased ANGPTL3 and found that ANGPTL3 blockade therapy is effective in cancer treatment and can synergistically enhance anti-PD1 therapeutic efficacy. These suggest that ANGPTL3 is a villainous molecule that suppresses antitumor immunity and a promising target molecule for developing not only biomarkers to predict potential anti-PD1 responses more accurately but also therapeutic drugs to enhance its therapeutic efficacy more effectively. Strategies targeting ANGPTL3 may contribute to improving clinical outcomes in anti-PD1/-PDL1 therapy for AGC.

Tumoral ANGPTL3 has been reported as a risk factor that promotes tumor proliferation, invasion, and angiogenesis, thereby adversely affecting patient prognosis in various types of cancer, including colorectal cancer (16), ovarian cancer (17), and cervical cancer (18). In gastric cancer, however, tumoral ANGPTL3 has been reported to play the opposite role, suppressing the proliferation and metastasis of gastric cancer cells (19). For example, ANGPTL3 expression in gastric cancer cells is suppressed by the methyltransferase METTL3 (20), and patients with low ANGPTL3 expression levels in gastric cancer tissues have a significant poor prognosis (21). However, open databases, such as ProteinAtlas (https://www.proteinatlas.org/), show that human tumor cell lines of gastrointestinal cancers, including gastric cancer and colorectal cancer, do not express ANGPTL3. Furthermore, we confirmed that ANGPTL3 was not detected by ELISA in the culture supernatant of several human gastric cancer cell lines and mouse tumor cell lines. These suggest that, in gastric cancer, ANGPTL3 may play a tumor-suppressive role only under specific conditions in which it is co-expressed with METTL3.

In contrast, other studies reported that ANGPTL3 increases specifically in the serum of patients with gastric cancer, and it is a potential biomarker that can identify and diagnose gastric cancer (22). This partly supports our findings that patients with high plasma ANGPTL3 levels had a significantly poorer prognosis after nivolumab therapy. However, no correlation was observed between the ELISA data and the proteomics data. Although the precise reasons are unclear, this may be due to differences in the principles, sensitivity, and specificity for quantifying proteins between the two methods. Particularly, proteomics simultaneously measures the expression of multiple proteins, which may be affected by other factors and/or interactions within the sample, leading to erroneous results due to cross-reactivity with other proteins. Therefore, ELISA is considered a more accurate analytic method than proteomics. Importantly, ANGPTL3 is known to exert its functions not by itself but through interactions with other factors and the formation of complexes. For example, ANGPTL3 produced in the liver does not immediately function in peripheral blood; it binds to ANGPTL8 at the N-terminus to form a complex, which then increases its affinity for lipoprotein lipase and other enzymes, enabling it to potently inhibit them (23). Therefore, clarifying other molecules that interact with or form complexes with ANGPTL3 will further strengthen the conclusions of this study and their clinical implications.

The significant anti-ANGPTL3 therapeutic efficacy observed in mouse tumor models raises hope that this therapy may be effective in clinical cancer therapy, especially for patients with increased ANGPTL3 in peripheral blood. Tumor-suppressive role of ANGPTL3 in inhibiting tumor growth and metastasis and activating NK cells to enhance antitumor immunity has also been reported, albeit in ovarian cancer (24). Another study reported that ANGPTL3 regulates the immune system by inhibiting the IL1β action through interfering with the interaction between IL1R1 and IL1RAP (25). However, IL1β plays a dual role in tumor immunity: it plays a tumor-promoting role by inducing tumor growth, invasion, metastasis, angiogenesis, and immunosuppression, whereas it also plays a tumor-suppressing role by enhancing antitumor immunity through T-cell activation (26, 27). Therefore, even though our study demonstrated induction of antitumor immunity by anti-ANGPTL3 therapy, the immunologic mechanism of ANGPTL3 action remains to be fully elucidated. Further studies to clarify this point will accelerate the clinical application of targeting ANGPTL3 in cancer therapy. Several drugs targeting ANGPTL3, such as evinacumab (28) and zodasiran (29), have already been developed in clinical therapy for familial hypercholesterolemia and mixed hyperlipidemia, albeit not for cancer. It is expected that the repositioning of these drugs, for which safety in humans has already been confirmed, to cancer treatment would enable more rapid and efficient development of new anticancer drugs.

However, this study has several limitations. First, because of the single-arm treatment setting without a control group, it is difficult to determine whether high ANGPTL3 levels are a general indicator of poor prognosis in AGC or whether they are specifically associated with unresponsiveness to anti-PD1 therapy. Second, the cutoff values determined in this study may overestimate the impact of each factor. To solidify the clinical significance of targeting ANGPTL3, further analysis using other statistical methods seems necessary. Third, because of the limited number of tumor sections available, we did not examine ANGPTL3 expression in tumor tissues, which may have had some effects on patient prognosis.

Nevertheless, this study provides significant evidence that peripheral ANGPTL3 is a significant risk factor for anti-PD1/-PDL1 therapy for AGC. Targeting ANGPTL3 in the peripheral circulation may be a promising strategy for improving clinical outcomes in anti-PD1/-PDL1 therapy for gastric cancer. Larger clinical studies with more patient samples would contribute to drawing more robust conclusions about the applicability of the results to clinical practice.

## Supplementary Material

Table S1 List of relationship between each marker level divided by the cutoff value and patient characteristics

Figure S1 Proteomic profiling ranking data (related to Figure 1)

Figure S2 Relationship between proteomic and ELISA data (related to Figure 1)

Figure S3 ANGPTL3 data by ELISA (related to Figure 4)

Figure S4 In vivo therapeutic experiments using other tumor models (related to Figure 4)

Figure S5 FCM gating strategy (related to Figure 4)

## Data Availability

Data are available upon request from the corresponding author. The proteomic data generated in this study are not publicly available because of lack of patient consent to deposit the data in a public repository but are available upon reasonable request from the corresponding author.
