# Supplementary material for: ANGPTL3 in the Peripheral Circulation Is Associated with Resistance to Anti-PD1 Therapy in Advanced Gastric Cancer
Source: Cancer Res Commun. 2026 Feb 19;6(2):350–8. doi: 10.1158/2767-9764.CRC-25-0793 (PMC13138227; doi:10.1158/2767-9764.CRC-25-0793)
Supplement: Table S1 — List of relationship between each marker level divided by the cutoff value and patient characteristics [file crc-25-0793_table_s1_suppst1.pdf]

Table S1. Relationship between each marker level divided by the cutoff value and patient characteristics (Age and the significant data only)

Time-Marker	Variable	Low	High	P values
Pre-Seq.10382-1	Average age	66	66	0.894
	Sex			0.023
	- Female	18.2%	9.1%	
	- Male	64.8%	8.0%	
	Lymph node metastasis			0.038
	- No	25.0%	10.2%	
	- Yes	58.0%	6.8%	
Pre-Seq.10391-1	Average age	66	67	0.953
	Sex			0.010
	- Female	17.0%	10.2%	
	- Male	64.8%	8.0%	
Pre-Protein	Average age	68	66	0.538
	Sex			
	- Female	20.9%	7.7%	
	- Male	64.8%	6.6%	
Post-Seq.10382-1	Average age	66	68	0.356
	Lymph node metastasis			0.012
	- No	21.3%	15.0%	
	- Yes	55.0%	8.8%	
Post-Seq.10391-1	Average age	66	68	0.356
	Lymph node metastasis			0.028
	- No	18.8%	17.5%	
	- Yes	48.8%	15.0%	
	ALP			0.032
	- < 350U/L	43.8%	12.5%	
	- ≥ 350 U/L	23.8%	20.0%	
Post-Protein	Average age	67	61	0.147
	NLR			0.020
	- < 1.93	51.3%	2.5%	
	- ≥ 1.93	35.0%	11.3%	

The values indicate the percentage of patients compared to the total number of patients.
