# Supplementary material for: ANGPTL3 in the Peripheral Circulation Is Associated with Resistance to Anti-PD1 Therapy in Advanced Gastric Cancer
Source: Cancer Res Commun. 2026 Feb 19;6(2):350–8. doi: 10.1158/2767-9764.CRC-25-0793 (PMC13138227; doi:10.1158/2767-9764.CRC-25-0793)
Supplement: Figure S1 — Proteomic profiling ranking data (related to Figure 1) [file crc-25-0793_figure_s1_suppsf1.pdf]

Proteins	Pre-treatment					Post-treatment				
	vs. Late PD	vs. Early PD	vs. Late PD	vs. Early PD	Late vs. Early	vs. Late PD	vs. Early PD	vs. Late PD	vs. Early PD	Late vs. Early
ANGPTL3-1	7.0	8.3	0.027	0.014	0.738	12.6	31.7	0.016	0.000	0.039
C2	3.1	4.6	0.008	0.032	0.400	2.4	4.9	0.014	0.001	0.046
DAB2	3.0	5.8	0.001	0.016	0.146	4.9	3.8	0.002	0.000	0.443
CFHR4	9.1	9.7	0.044	0.030	0.913	7.7	17.4	0.048	0.006	0.139
HSPE1	3.1	4.0	0.000	0.004	0.353	3.2	5.3	0.039	0.009	0.252
HSPA1A-1	1.3	1.9	0.001	0.016	0.121	1.9	2.0	0.050	0.000	0.927
IL18	2.0	3.1	0.026	0.018	0.263	2.4	3.4	0.004	0.000	0.139
LYN	3.0	3.2	0.004	0.014	0.839	4.3	3.2	0.000	0.015	0.232
MAPRE3	4.3	4.0	0.008	0.042	0.881	5.1	5.9	0.030	0.022	0.770
PPBP	3.6	4.4	0.000	0.000	0.360	2.9	3.2	0.001	0.001	0.676
SPARC	2.9	4.6	0.031	0.012	0.209	6.8	10.1	0.001	0.000	0.228
STAT3	1.9	3.1	0.019	0.012	0.119	2.4	2.6	0.004	0.000	0.712
TAPBPL	2.9	3.4	0.010	0.006	0.604	2.5	8.3	0.009	0.015	0.052
FLT4	1.9	2.1	0.025	0.028	0.708	2.5	5.1	0.008	0.004	0.069

Figure S1. Proteins that were significantly more than two-fold higher in early/late PD groups compared to those of the nonPD group in proteomic profiling of plasma obtained from AGC patients receiving nivolumab therapy (related to Figure 1)

The heatmap shows the ratio of the mean of early/late PD groups to the mean of the NonPD group, and is color-coded from 1 to the maximum value at each time point. P values analyzed by the Mann-Whitney test are shown to the right of the heatmap.
