# Supplementary material for: ANGPTL3 in the Peripheral Circulation Is Associated with Resistance to Anti-PD1 Therapy in Advanced Gastric Cancer
Source: Cancer Res Commun. 2026 Feb 19;6(2):350–8. doi: 10.1158/2767-9764.CRC-25-0793 (PMC13138227; doi:10.1158/2767-9764.CRC-25-0793)
Supplement: Figure S2 — Relationship between proteomic and ELISA data (related to Figure 1) [file crc-25-0793_figure_s2_suppsf2.pdf]

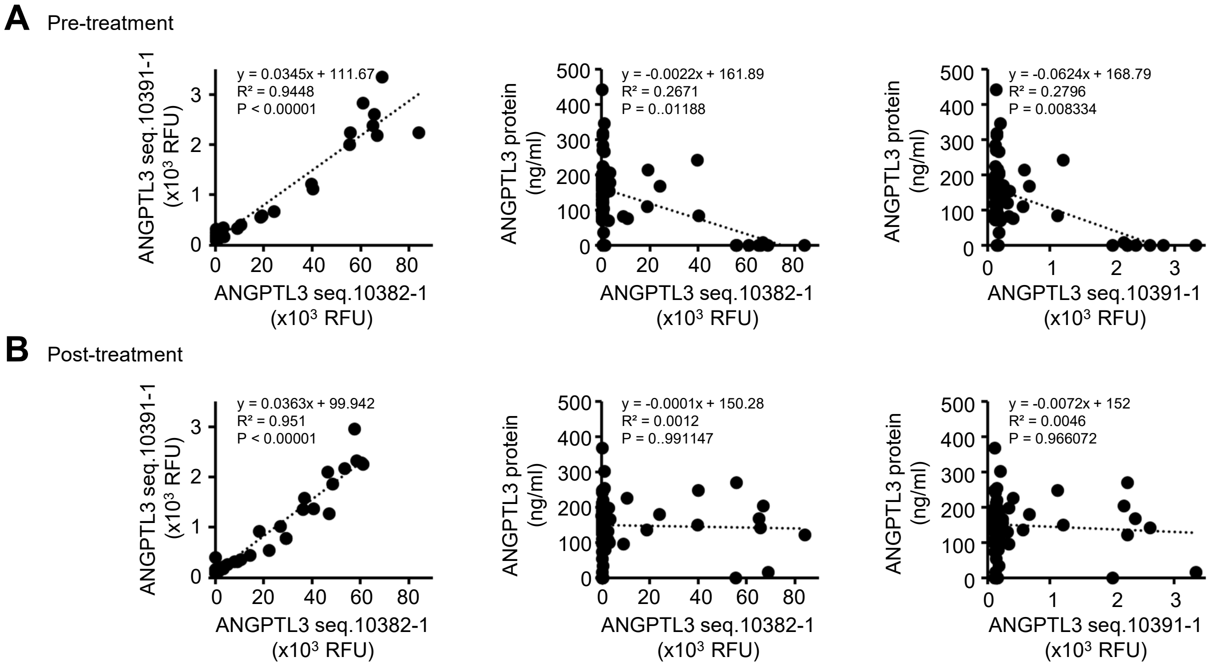

Figure S2. Relationship between ANGPTL3 data analyzed by proteomic profiling and ELISA (related to Figure 1)

“ANGPTL3 seq.10382-1” and “ANGPTL3 seq.10391-1” are data from proteomic profiling using using the 7k SomaScan v4.1. “ANGPTL3 protein” is ELISA data.

(A) Pre-treatment.

(B) Post-treatment.
