# Supplementary material for: ANGPTL3 in the Peripheral Circulation Is Associated with Resistance to Anti-PD1 Therapy in Advanced Gastric Cancer
Source: Cancer Res Commun. 2026 Feb 19;6(2):350–8. doi: 10.1158/2767-9764.CRC-25-0793 (PMC13138227; doi:10.1158/2767-9764.CRC-25-0793)
Supplement: Figure S3 — ANGPTL3 data by ELISA (related to Figure 4) [file crc-25-0793_figure_s3_suppsf3.pdf]

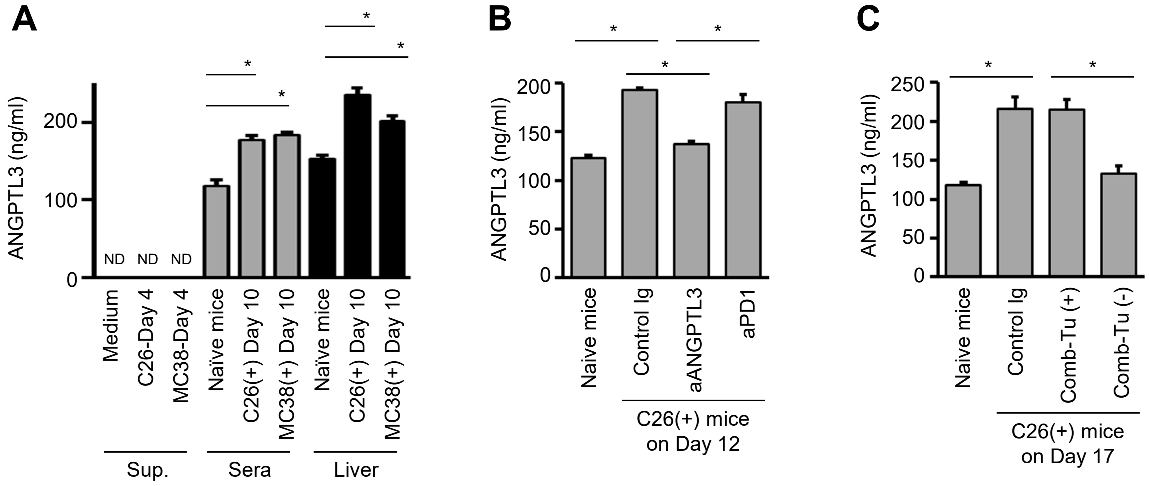

Figure S3. ANGPTL3 is not produced by tumor cells, but is derived from the host (related to Figure 4)

In the in vitro setting, murine colorectal cancer Colon26 (C26) and MC38 cells as a reference were cultured in 10%FCS-containing DMEM medium for 4 days, and the supernatants were harvested for assays. In the in vivo setting, BALB/c mice were subcutaneously (5×10^5) implanted with Colon26 cells, and received intraperitoneal injection with anti-ANGPTL3 mAb, anti-PD1 mAb, and/or mouse IgG as a control at 10 mg/kg on days 3 and 10 after tumor implantation. On day 10, 12 or 17, peripheral blood and liver were harvested from the mice for assays. PBS-perfused livers were homogenized in 10 ml of PBS on ice, the suspension was centrifuged at 3000 rpm for 10 min, and the supernatant was filtered through a filter (0.45 μm). The tumor cultured supernatants, sera, and liver extract solution were tested for mouse ANGPTL3 using an ELISA kit (R&D #MANL30). (A) ANGPTL3 is produced by liver of mice, but not by tumor cells (n = 8). ND, not detected. (B) ANGPTL3 is further increased in the peripheral blood of mice after tumor implantation, but its levels were significantly reduced by treatment with anti-ANGPTL3 mAb (n = 8). (C) ANGPTL3 levels are significantly low in mice cured by anti-ANGPTL3/PD1 combination therapy (n = 8). In three repeated experiments, 30 mice that had received the combination therapy were divided into two groups: those that had disappeared (designated Tu(-)) and those that still had tumors (designated Tu(+), 8 mice were randomly selected). Graphs show means ± SDs. *$P < 0.001$ by Mann-Whitney U test.
