# Supplementary material for: ANGPTL3 in the Peripheral Circulation Is Associated with Resistance to Anti-PD1 Therapy in Advanced Gastric Cancer
Source: Cancer Res Commun. 2026 Feb 19;6(2):350–8. doi: 10.1158/2767-9764.CRC-25-0793 (PMC13138227; doi:10.1158/2767-9764.CRC-25-0793)
Supplement: Figure S4 — In vivo therapeutic experiments using other tumor models (related to Figure 4) [file crc-25-0793_figure_s4_suppsf4.pdf]

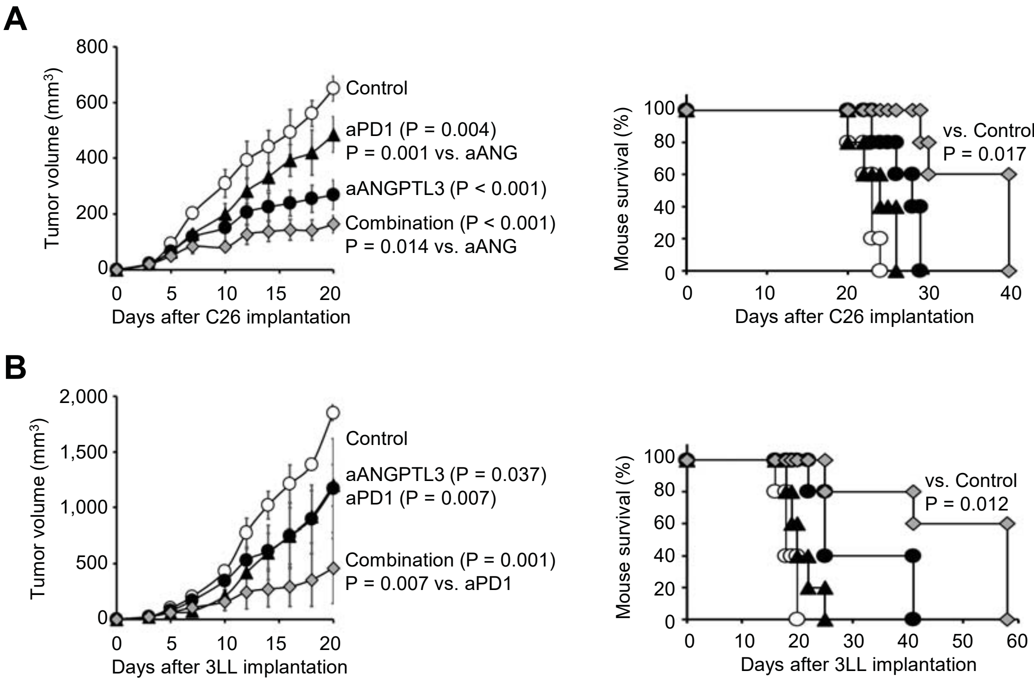


**Figure S4. ANGPTL3 is not produced by tumor cells, but is derived from the host (related to Figure 4)**

Mice received intraperitoneal injection with anti-ANGPTL3 mAb, anti-PD1 mAb, and/or mouse IgG as a control at 10 mg/kg on days 3 and 10 after tumor implantation (n = 5). (A) Anti-tumor effects in mouse colorectal cancer ascites model. BALB/c mice were subcutaneously ($1 \times 10^6$) and intraperitoneally ($5 \times 10^5$) implanted with Colon26 cells. (B) Anti-tumor effects in mouse lung cancer metastasis model. C57BL/6 mice were subcutaneously ($1 \times 10^6$) and intravenously ($5 \times 10^5$) implanted with 3LL cells. Bar graphs show means ± SDs. P values by Mann-Whitney U test.
