# Supplementary material for: ANGPTL3 in the Peripheral Circulation Is Associated with Resistance to Anti-PD1 Therapy in Advanced Gastric Cancer
Source: Cancer Res Commun. 2026 Feb 19;6(2):350–8. doi: 10.1158/2767-9764.CRC-25-0793 (PMC13138227; doi:10.1158/2767-9764.CRC-25-0793)
Supplement: Figure S5 — FCM gating strategy (related to Figure 4) [file crc-25-0793_figure_s5_suppsf5.pdf]

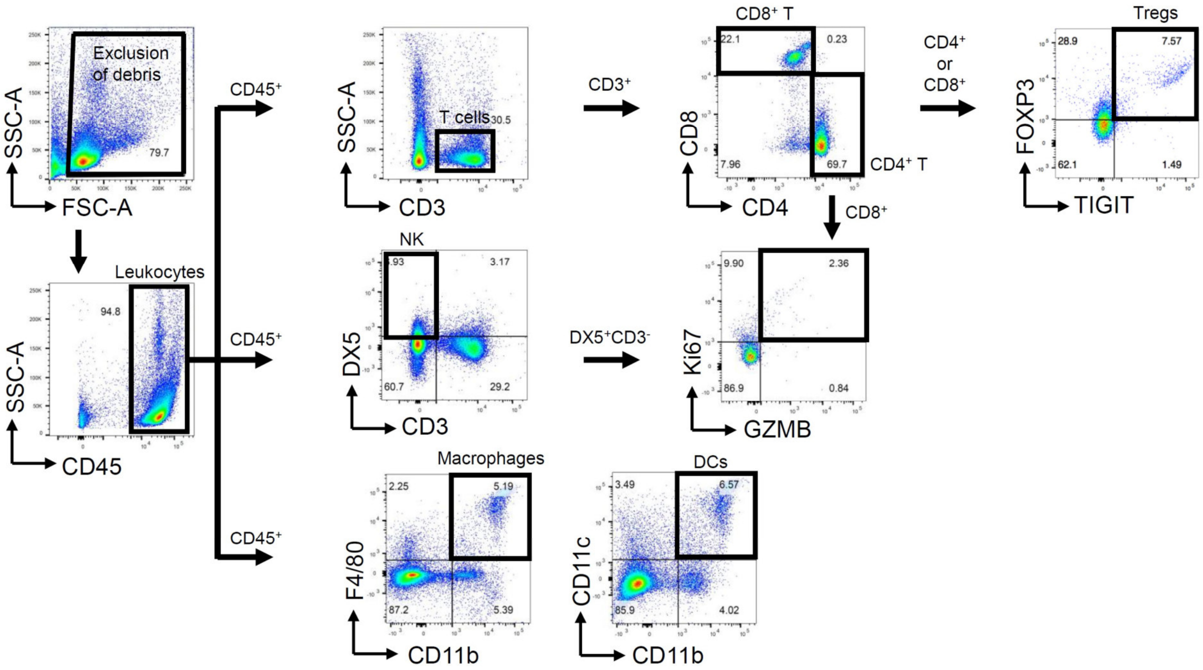

Figure S5. Gating strategy for flow cytometric analysis of mouse samples (related to Figure 4)

Debris was firstly excluded by gating FSC/SSC, and the specific cell populations were defined by comparing to the isotype controls after gating $CD45^{+}$ leukocytes. Here, dot plot panels were developed using spleen cells, which have many cells and displays many dots, in order to clearly show the validity of the axis that separates the negative and positive cell fractions.
